# The effects of postbiotics and glycyrrhetinic acid on immune response and inflammation-related genes during *H. pylori* eradication therapy

**DOI:** 10.1186/s12866-025-04191-1

**Published:** 2025-12-13

**Authors:** Erhan Tek, Nizami Duran, Elif Yaprak Colak, Gulay Gulbol Duran, Tuncer Kutlu, Hamdullah Suphi Bayraktar, Sibel Dagli, Mehmet Demir

**Affiliations:** 1 Department of Medical Microbiology, Medical Faculty, Hatay Mustafa Kemal University, Antakya-Hatay, 31060 Türkiye; 2 Department of Medical Biology, Medical Faculty, Hatay Mustafa Kemal University, Antakya-Hatay, 31060 Türkiye; 3 Department of Pathology, Veterinary Faculty, Hatay Mustafa Kemal University, Antakya-Hatay, 31060 Türkiye; 4 Department of Gastroenterology, Medical Faculty, Hatay Mustafa Kemal University, Antakya-Hatay, 31060 Türkiye

**Keywords:** *H.pylori*, Postbiotics, *S.thermophilus*, *L.casei*, AGS cell line, *Glycyrrhetinic acid*, Inflammation-related gene expression, Microbiological eradication

## Abstract

**Aim:**

This study aims to test the effectiveness of postbiotics from *Streptococcus thermophilus* and *Lactobacillus casei*, along with *Glycyrrhetinic acid*, against *H. pylori* in both AGS cells (a human gastric adenocarcinoma cell line) and an experimental rat gastritis model.

**Method:**

The effectiveness of the compounds (postbiotics and *G. acid*) against *H. pylori* was evaluated by measuring the expression levels of various genes, including NF-κB, IL-1β, IL-6, IL-8, IL-10, TNF-α, COX-2, FOX-M1, and IL-33 in AGS cells using the Real-Time PCR method. Additionally, bacterial loads in gastric tissue were quantified using culture-based colony-forming unit (CFU) analysis and expressed as log₁₀ CFU/g. The study also evaluated the effectiveness of combinations of these components in the rat gastritis model through microbiological and histopathological analyses.

**Results:**

The postbiotics of *S. thermophilus* and *L. casei* have shown a significant reduction in the expression level of NF-κB when compared to the control group. The *S. thermophilus* plus *L. casei* treatment group showed a 10-fold decrease in NF-κB expression, while the *G. acid* plus *L. casei* treatment group showed a 5-fold decrease. TNF-α expression levels were also lower in the *S. thermophilus* and *L. casei* treatment groups with 10-fold and 5-fold reductions, respectively. The expression of IL-1β was significantly reduced in all treatment groups compared to the control group, with a 10-fold decrease in expression. The *S. thermophilus* plus *L. casei* treatment group exhibited a 10-fold reduction in IL-6 release, a proinflammatory cytokine. Similarly, the expression of IL-8 was reduced by 10-fold and 2-fold in the *S. thermophilus* and *L. casei* treatment groups, respectively. Additionally, bacterial loads in gastric tissue were quantified using culture-based colony-forming unit (CFU) analysis and expressed as log₁₀ CFU/g. The mean bacterial load in the infected control group was 6.14 ± 0.18 log₁₀ CFU/g, while the quaternary treatment group (*S. thermophilus* + *L. casei* + *G. acid* + antibiotic) achieved complete eradication with no detectable colonies (0.00 ± 0.00 log₁₀ CFU/g). Ternary and binary treatment groups exhibited intermediate bacterial loads, ranging from approximately 1.63 ± 0.09 to 3.24 ± 0.17 log₁₀ CFU/g, indicating partial efficacy compared to the control group.

**Conclusion:**

These promising findings can pave the way for the development of novel therapeutic approaches for treating *H. pylori*-associated gastritis. The combination of molecular (gene expression) and microbiological (CFU) data demonstrates the multi-dimensional efficacy of postbiotics and glycyrrhetinic acid as potential adjunctive agents.

**Supplementary Information:**

The online version contains supplementary material available at 10.1186/s12866-025-04191-1.

## Introduction

*Helicobacter pylori* is a Gram-negative, spiral-shaped, microaerophilic bacterium that infects over half of the global population, contributing to chronic gastritis, peptic ulcers, and stomach cancer [1]. *H. pylori* is treated with antibiotics and PPIs, but antibiotic overuse has led to resistance and treatment failures. Long-term antibiotic use also causes adverse effects, prompting research into alternative therapies and improved drug efficacy [2, 3].

Most drug research has recently been conducted on natural or herbal products due to their low cytotoxic effects. One such plant commonly used for treating stomach ailments is *Glycyrrhiza glabra*, which contains *Glycyrrhizic acid*, a potent ingredient that can be hydrolyzed into *Glycyrrhetinic acid*. *Glycyrrhetinic acid* has demonstrated antimicrobial and anti-inflammatory effects, and previous studies reported its efficacy against *H. pylori* in vitro. Its inclusion in this study aimed to explore its synergistic potential when combined with postbiotics and antibiotics. Furthermore, *Glycyrrhetinic acid* has been reported to suppress pro-inflammatory cytokines (e.g., IL-1β, TNF-α) and inhibit NF-κB pathway activation, both of which are critical in *H. pylori*-induced gastric inflammation and carcinogenesis. The rationale for including *G. acid* in this study was twofold: (1) to explore its potential synergistic effects when combined with postbiotics and antibiotics, aiming to enhance eradication rates while mitigating inflammation, and (2) to evaluate its therapeutic relevance in addressing antibiotic resistance by contributing a natural anti-*H. pylori* and anti-inflammatory agent to combination therapy. This approach aligns with recent literature emphasizing the role of phytochemicals in combating antibiotic-resistant pathogens and improving host immune response [4–7]. Various studies have reported that *Glycyrrhiza glabra* exhibits antibacterial [8], antiviral [4], anti-inflammatory [5], antiulcer [9], antiallergic [10], anti-oxidant [11], and anti-tumoral [12] properties. However, the effectiveness of *G.acid* against *H. pylori*, a bacteria responsible for causing stomach ulcers, has only been tested in a limited number of studies [13, 14]. In a previous study, *G.acid* showed significant antimicrobial activity against *H. pylori in vitro* [6]. Conducting in vivo studies will help clarify the effectiveness of *G.acid* against *H. pylori* and determine its potential for treating stomach ulcers [15, 16].

Some studies suggest that supplementing with certain strains of probiotics may help improve eradication rates when antibiotic therapy is not effective enough for treating *H. pylori* infection [13]. While some probiotics may have toxic effects, many are well-tolerated and produce bioactive compounds with antimicrobial and immune-modulating properties. They enhance host resistance to pathogens, regulate gut microbiota, and influence immune responses by modulating cytokine expression [17–21].

Probiotics produce bioactive substances such as short-chain fatty acids, enzymes, proteins, organic acids, vitamins, and amino acids. These substances are also known as postbiotics. The cell wall components of these bacteria also have similar health effects. Postbiotics are compounds that are produced by probiotic bacteria. They contain soluble factors such as various enzymes, proteins, polysaccharides, short-chain fatty acids, and peptidoglycans. These compounds have immunomodulatory and anti-inflammatory effects on the host, improving their health and well-being [22, 23].

Lactobacillus and Streptococcus postbiotics, composed of secreted metabolites such as short-chain fatty acids, exopolysaccharides, and bacteriocins, have been reported to be effective in boosting cell-mediated immunity and reducing inflammation. While both strains share core components, *S. thermophilus*-derived postbiotics are rich in exopolysaccharides, whereas *L. casei* postbiotics are known for their immunoregulatory proteins and higher peptidoglycan content, which may explain the variation in their anti-inflammatory activity. Lactobacillus species produce postbiotics that play a vital role in supporting the immune system of the intestinal mucosa. In one study, the potential effect of postbiotic fractions of intestinal *L. rhamonosus* and *L. plantarum* on the immune response induced by pro-inflammatory stimuli was investigated. The study reported that the presence of probiotic bacterial components on the mucosal surface during the initial and final stages of inflammatory conditions results from cellular interactions that regulate inflammation and prevent damage to the intestinal epithelium. Postbiotics of *L.rhamnosus* and *L.plantarum* have been shown to activate *IL-10*, an anti-inflammatory cytokine, and reduce inflammation by regulating the *IL-18*-related response [24, 25].

Studies have indicated that infants who consume infant formula containing postbiotic products derived from *S.thermophilus* experience less severe acute diarrheal attacks, an increase in thymus size, a decrease in fecal calprotectin, and an increase in secretory IgA. Furthermore, *S.thermophilus* postbiotics stimulate immunomodulatory reactions in dendritic cells by increasing the release of *IL-10* and stimulating the Th1 immune system. Although the advantageous properties of postbiotics from probiotic bacteria are well established, the mechanisms underlying their interaction with immune cells and their ability to affect immune modulation require further investigation. Moreover, the precise molecular mechanism that enables postbiotics to produce positive effects on the host is yet to be fully comprehended [26–29].

Probiotics, mainly lactic acid bacteria like *Lactobacillus* and *Streptococcus*, modulate gut microbiota and exhibit antimicrobial activity. *S. thermophilus* exo-polysaccharides may reduce *H. pylori*-induced inflammation, but further studies are needed. *Lactobacillus* strains show anti-*H. pylori* effects and enhance immune responses by modulating T-cell balance and stimulating phagocytosis, chemokine, and cytokine production [18, 30–36].

Scientific research on the activities of probiotic microorganisms against *H. pylori* remains limited. This study aimed to investigate the role of postbiotics derived from probiotic microorganisms in improving *H. pylori* treatment outcomes. Following a comprehensive review of the literature, although some previous studies addressing this topic exist, to the best of our knowledge, this is the first study to evaluate the combined therapeutic potential of antibiotics, *S. thermophilus* and *L. casei* postbiotics, and glycyrrhetinic acid in both in vitro and in vivo models. The findings of this study may serve as a valuable reference for future research in this field.

The efficacies of combinations of *G.acid*, *S.thermophilus*, and *L.casei*’s postbiotics against *H.pylori* were evaluated in AGS cells (human gastric adenocarcinoma cell line) and a rat model of gastritis induced by *H.pylori*. The expression levels of *NF-kB*,* IL-1B*,* IL-6*,* IL-8*,* IL-10*,* TNF-α*,* COX-2*,* FOX-M1*, and *IL-33* genes were determined in AGS cells. Then, the efficacies of these components in the rat gastritis model experimentally induced with *H.pylori* were compared with the standard antibiotic treatment groups. Microbiological and histopathological techniques were used for in-vivo evaluations.

## Materials & methods

The effects of *G.acid*, *S. thermophilus*, and *L. casei* postbiotics on *H. pylori* were assessed in AGS cells and a rat gastritis model. Gene expression levels (NF-κB, IL-1B, IL-6, IL-8, IL-10, TNF-α, COX-2, FOX-M1, IL-33) were analyzed in AGS cells. In-vivo efficacy was compared with standard antibiotic treatment using microbiological and histopathological methods.

### *H. pylori *culture

*H. pylori* (NTCC 11637) was obtained from Ankara Refik Saydam Hygiene Institute. Strains were incubated in Brucella Broth with 5% FBS under microaerophilic conditions for five days, then passaged onto 5% sheep blood agar and incubated at 37 °C for seven days. Strains were sensitive to Amoxicillin and Clarithromycin, with susceptibility tests performed before experiments [37].

### Postbiotics of *L. casei* and *S. thermophilus*

*L. casei* and *S. thermophilus* were cultured on MRS agar at 37 °C under anaerobic conditions. Supernatants were collected by centrifugation (6000 rpm, 30 min, 4 °C) and filtered (0.45 μm). Blank MRS medium was processed similarly as a control.

### Glycyrrhetinic acid and antibiotics

*G.acid* (Sigma-Aldrich, USA) was dissolved in 0.1% DMSO (Merck) for Vero cells. Amoxicillin (Deva, TR) and Clarithromycin (Abbott, USA) were used as standard drugs. H. pylori susceptibility was tested before the study (MIC: Amoxicillin 0.10 µg/mL, Clarithromycin 0.20 µg/mL) [37].

### Cell culture

Vero (ATCC CCL-81) and AGS (ATCC CRL-1739) cell lines were used as healthy and H. pylori-infected models, respectively. Cells were cultured in RPMI-1640 with 10% FBS, HEPES, penicillin/streptomycin, and glutamine at 37 °C, 5% CO₂. Non-toxic doses of postbiotics and *G.acid* were determined in Vero cells. Cell density was set at 1 × 10⁶ cells/mL for experiments.

AGS cells were infected with *H. pylori* (1 × 10⁸ CFU/mL, MOI 100) for 6 h at 37 °C with 5% CO₂. Incubation continued for 96 h to assess the effects of postbiotics and *G.acid* on intracellular bacteria. Cells were collected at specific time points for PCR analysis.

### Cytotoxicity tests

Cytotoxicity was evaluated using Vero cells in 96-well plates (1 × 10⁶ cells/mL) with RPMI 1640 + 10% FBS. Compounds were dissolved in 0.1% DMSO, which served as a negative control. Non-toxic concentrations of G.acid, S. thermophilus, and L. casei postbiotics were determined by the MTT assay [38].

### MTT method

The MTT assay, a standard method for assessing cell viability and cytotoxicity, was used to evaluate the effects of postbiotics, G.acid, and drugs (Lansoprazole, Amoxicillin, Clarithromycin) on Vero cells. Cells (1 × 10⁵/well) were seeded in 96-well plates and incubated at 37 °C with 5% CO₂ for 24 h. Serial dilutions of postbiotics (2.5–20.0 µg/mL) and *G.acid* (0.25–2.0 µg/mL) were added. After incubation, MTT solution was applied for 2 h, followed by DMSO to dissolve formazan. Absorbance at 570 nm was measured using a spectrophotometer. IC50 values were determined from dose-response curves, and cytotoxicity was calculated as test/control absorbance ×100. Experiments were repeated three times per concentration.

### Activity studies

Non-toxic doses of postbiotics (12.5 µg/mL) and G.acid (1.25 µg/mL) were determined using Vero cells. Cell viability and morphology were monitored daily under an inverted microscope. Cells (1 × 10⁵/mL) were incubated at 37 °C with 5% CO₂, treated with test compounds, and incubated for 96 h. After trypsinization and centrifugation, viability was assessed via hemocytometer, and mRNA expression levels were analyzed by RT-PCR.

### Infection of AGS cells with *H. pylori*

AGS cells were cultured in DMEM with 4 mM L-glutamine, 10% FBS, and antibiotics. For gene expression studies, antibiotic-free medium was used. The effects of postbiotics, G.acid, and their combinations on NF-κB, IL-1B, TNF-α, FOX-M1, IL-6, IL-8, IL-10, and IL-33 gene expression were evaluated against an untreated control group (Table 1).

**Table 1:** Experimental groups and numbers in gene expression study

Groups	The number of repetitions
Group 1 (HP group)	3
Group 2 (STP group)	3
Group 3 (GA group)	3
Group 4 (LCP group)	3
Group 5 (AB group	3
Group 6 (STP + GA group)	3
Group 7 (STP + LCP group)	3
Group 8 (GA + LCP group)	3
Group 9 (GA + AB group)	3
Group 10 (STP + LCP + AB group)	3
Group 11 (STP + GA + LCP group)	3
Group 12 (STP + GA + LCP + AB group)	3

*HP: H.pylori*

*STP: S.thermophilus’ postbiotics*

*GA: Glysithetinic acid*

*LCP: L.casei’s postbiotics*

*AB: (Amoxicillin + Clarithromycin)*

### RNA isolation

Total RNA was isolated from AGS cells using High-Pure RNA Tissue Kits (Thermo Fisher Scientific, USA). DNase I treatment was applied to eliminate DNA contamination. RNA integrity was confirmed via agarose gel electrophoresis. cDNA was synthesized using the Thermo Scientific cDNA Synthesis Kit and stored at −20 °C until use.

### RT-qPCR

Gene expression levels of NF-κB, IL-1B, IL-6, IL-8, IL-10, TNF-α, COX-2, FOX-M1, and IL-33 were analyzed using SYBR Green-based RT-qPCR on the Montana 4896 system (Anatolia, Türkiye). PCR conditions included initial denaturation at 95 °C for 10 min, followed by 35 cycles of 95 °C for 15 s and 60 °C for 60 s, with a final step at 99.9 °C for 10 min. GAPDH was used for normalization, and relative expression levels were calculated using the 2^−∆∆Ct^ method. Primer sequences are provided in Table 2.

**Table 2:** Oligonucleotide sequences were used in the present study

Primers	Sequences	References
GAPDH	5‘-TGCACCACCAACTGCTTAGC-3‘	31
	5‘-GGCATGGACTGTGGTCATGAG-3’	
IL-33	5’-GGAAGAACACAGCAAGCAAAGCCT-3’	32
	5’-TAAGGCCAGAGCGGAGCTTCATAA-3’	
FOX-M1	5’-TGCAGCTAGGGATGTGAATCTTC-3’	33
	5’-GGAGCCCAGTCCATCAGAACT-3’	
COX-2	5′-CTTGCTGTTCCCACCCATGTCAAA-3′	34
	5′-TGCACTGTGTTTGGAGTGGGTTTC-3′	
*TNF-alpha*	5′-CGAGTGACAAGCCTGTAGC-3′	35
	5′-GGTGTGGGTGAGGAGCACAT-3′	
IL-10	5′-GTGATGCCCCAAGCTGAGA-3′	36
	5′-CACGGCCTTGCTCTTGTTTT-3′	
IL-6	5′-AAATGCCAGCCTGCTGA CGAAC-3′	34
	5′-AACAACAATCTGAGGTGCCCATGCTAC-3′	
IL-1 beta	5’-AAGCCCTTGCTGTAGTGGTG-3’	37
	5’-GAAGCTGATGGCCCTAAACA-3’	
IL-8	5’-AGCACTCCTTGGCAAAACTG-3’	38
	5’-CGGAAGGAACCATCTCACTG-3’	
Nf-kB	5’-AAAGACACATCCGGACCTCG-3’	39
	5’-TGTAAGAGTTCCCCTCCGGT-3’	

In-vivo tests

This study was approved by the Hatay Mustafa Kemal University Animal Ethics Committee (27.12.2018, No: 2018/11 − 9) and conducted at the university’s Experimental Research Center. All animals used in this study were sourced directly from the Experimental Research Center of Hatay Mustafa Kemal University. The animals were bred and maintained under institutional care and were not privately owned by any external institution, individual, or farm. Therefore, no external informed consent was required.

Male albino Wistar rats were randomly divided into 13 groups (Table 3) and housed under standard conditions (23 ± 2 °C, 12 h light/dark cycle) with ad libitum access to standard feed and water. A total of 65 male Wistar albino rats (~ 300 g) were used. Rats were inoculated with *H. pylori* (5 × 10⁸ cfu/mL, 1 mL/rat, twice daily for 3 days). After 15 days, colonization and gastritis were confirmed by histopathological and microbiological analysis. Rats received a standard eradication regimen (amoxicillin 50 mg/kg, clarithromycin 25 mg/kg, lansoprazole 20 mg/kg). Safe, non-toxic doses of postbiotics (*S. thermophilus*,* L. casei* at 12.5 µg/mL) and *G. acid* (1.25 µg/mL) were determined based on rat weight. Daily doses were 3.75 mL/rat for postbiotics and 0.375 mL/rat for *G. acid*, administered via gavage [39].

**Table 3:** Animal experiments treatment groups and numbers

Experimental groups	Numbers of rats
Group 1 (HP group)	5
Group 2 (Healthy control group)	5
Group 3 (STP treatment group)	5
Group 4 (GA treatment group)	5
Group 5 (LCP treatment group)	5
Group 6 (AB group)	5
Group 7 (STP + GA group)	5
Group 8 (STP + LCP group)	5
Group 9 (GA + LCP group)	5
Group 10 (GA + AB group)	5
Group 11 (STP + LCP + AB group)	5
Group 12 (STP + GA + LCP group)	5
Group 13 (STP + GA + LCP + AB group)	5
Total number of animals	65

*HP*: *H.pylori*

*STP*: *S.thermophilus*’ postbiotics

*GA*: *Glysithetinic acid*

*LCP*: *L.casei*’s postbiotics

*AB*: *(Amoxicillin + Clarithromycin)*

The ulcer index was measured by considering the following criteria as stated in the literature [40]. For this, the number and severity of the lesions were evaluated. The following scores were used for evaluation: I: Mild; II: Medium, the presence of edema, hyperemia, and single petechiae; III: The presence of submucosal hemorrhagic lesions with small erosions; IV: The presence of hemorrhagic lesions with severe erosions. Ulcer Index (UI) = (nI) + (nII) × 2 + (nIII) × 3/Number of animals (“n” is the number of lesions). Then, the indicated treatments were applied to the following treatment groups for ten days. Following the development of gastritis, the compounds and their various combinations were administered at 1 mL per rat per day. The treatment was orally administered daily during the ten-day treatment period.

At the end of the experimental procedures, all animals, including those in the control groups, were humanely sacrificed under deep anesthesia. A combination of ketamine (150 mg/kg) and xylazine (9 mg/kg) was administered intraperitoneally (i.p.) to ensure adequate anesthesia and unconsciousness before sacrifice. The selected dosages and administration route were based on widely accepted guidelines for laboratory rodents, aiming to minimize animal pain and distress. Following confirmation of deep anesthesia (loss of righting reflex and pedal withdrawal reflex), rats were sacrificed by exsanguination through cardiac puncture. This euthanasia protocol was reviewed and approved by the Hatay Mustafa Kemal University Animal Ethics Committee (Approval No: 2018/11 − 9), by institutional guidelines, and international ethical standards for animal research. This ensured that all procedures complied with ARRIVE guidelines and relevant national legislation.

Following the sacrifice, the rats’ gastric tissues were removed. Then, the tissue samples from rats were placed in PBS for microbiological analysis, in formaldehyde for histopathological analysis, and sent to laboratories.

### Microbiological analysis

Following euthanasia, gastric tissues were aseptically excised and immediately transferred into 10 mL of cold phosphate-buffered saline (PBS). One-gram tissue samples were homogenized and plated onto blood agar supplemented with 5% sheep blood. Plates were incubated at 37 °C for 72 h under microaerophilic conditions. Colonies were counted and expressed as log₁₀ CFU per gram of tissue. Each experimental group was analyzed in triplicate using independently treated animals.

In the infected control group, the mean bacterial load was 6.14 ± 0.18 log₁₀ CFU/g tissue (individual values: 6.32, 5.97, 6.13 log₁₀ CFU/g). No bacterial growth was detected in the negative control group (0.00 ± 0.00 log₁₀ CFU/g).

For treatment groups, the *G. acid* + Antibiotic (Binary-3) group had 3.24 ± 0.17 log₁₀ CFU/g (3.10, 3.20, 3.43), the *L. casei* + Antibiotic (Binary-2) group had 3.02 ± 0.07 log₁₀ CFU/g (3.02, 3.10, 2.95), and the *S. thermophilus* + Antibiotic (Binary-1) group had 2.83 ± 0.17 log₁₀ CFU/g (2.80, 3.02, 2.68). The ternary combination group (*S. thermophilus* + *L. casei* + Antibiotic) showed 1.63 ± 0.09 log₁₀ CFU/g (1.55, 1.73, 1.61), while the quaternary combination group (*S. thermophilus* + *L. casei* + *G. acid* + Antibiotic) exhibited complete eradication with no detectable colonies (0.00 ± 0.00 log₁₀ CFU/g).

Intermediate bacterial loads were observed in the binary and ternary treatment groups, while the quaternary group demonstrated total bacterial eradication. Infection rates were statistically evaluated using chi-square (χ²) tests (*n* = 5 per group), while CFU count comparisons among groups were analyzed using one-way ANOVA followed by Tukey’s post hoc test [41].

### Pathological evaluation

Gastric tissues were fixed in 10% neutral buffered formalin, washed, dehydrated in graded alcohols, cleared with xylene, and embedded in paraffin. Section (5 μm) were cut, deparaffinized, rehydrated, and stained with Hematoxylin and Eosin (H&E). Tissue images were captured using a light microscope (Olympus CX31). Gastritis severity was assessed semi-quantitatively as mild, moderate, or severe [42].

#### Statistical analysis

Data were analyzed using GraphPad Prism v10. Results are expressed as mean ± SD from at least three experiments. Statistical comparisons were made using one-way ANOVA with Tukey’s post hoc test or unpaired two-tailed t-tests where appropriate. Differences were considered significant at *p* < 0.05. Significance levels: *p* < 0.05 (*)*,* < 0.001 (****)***,*** < 0.0001 (***), < 0.00001 (****), and ns for non-significant results.

## Results

**Fig. 1:**
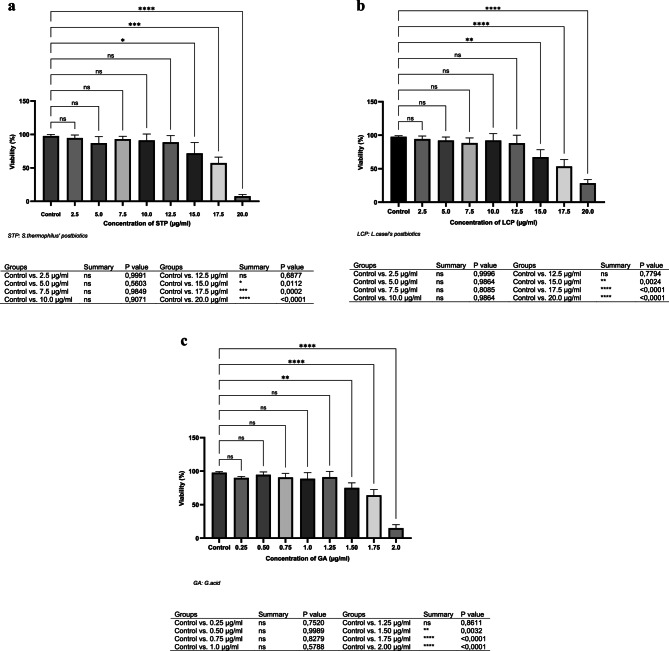
**a**-**c**. Cytotoxicity results of the *S.thermophilus*' (a) and *L.casei*’s (b) postbiotics, and *G.acid* (c) on the viability of Vero cells by MTT method

**Fig. 2:**
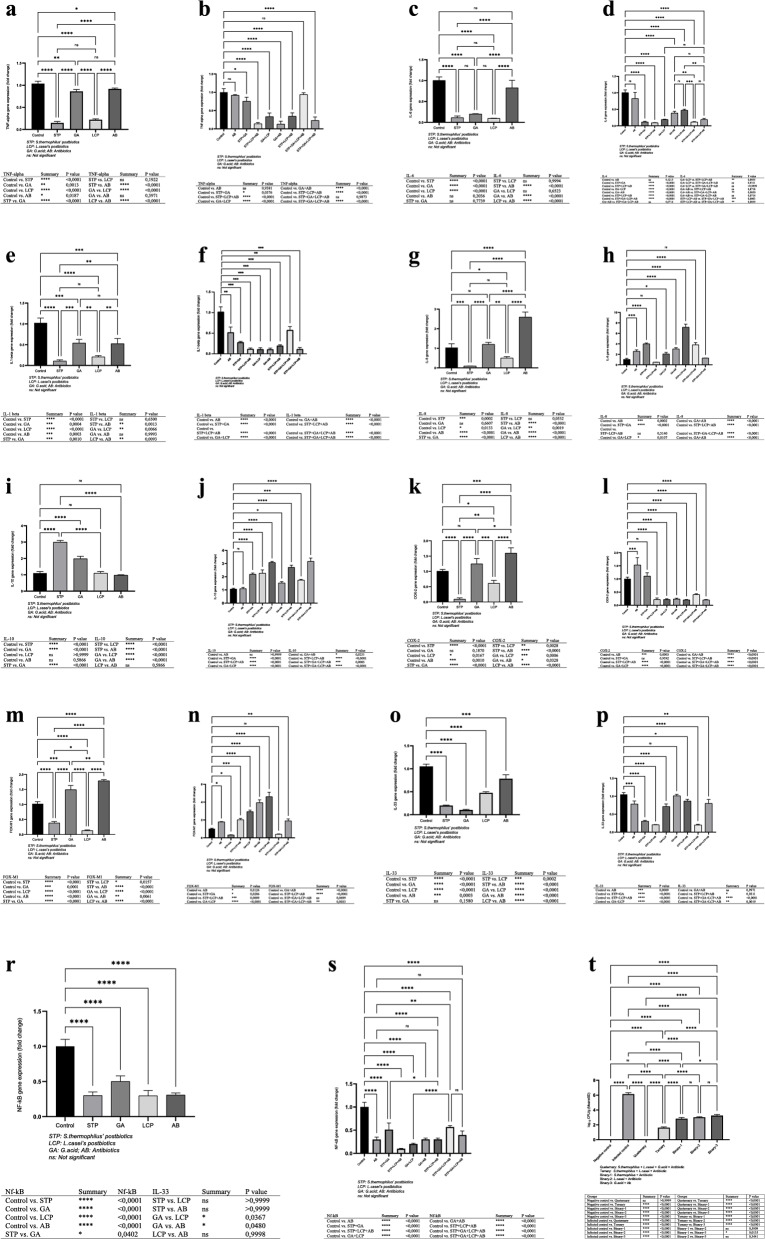
**a**. Effects of *G. acid*, *S. thermophilus,* and *L.casei*’s postbiotics on *TNF-alpha* expression on *H.pylori*-infected AGS cells. **b.** Synergistic effects of different combinations of *G.acid*,*S. thermophilus*, and *L.casei*’s postbiotics with the antibiotic group on *TNF-alpha* expression in *H.pylori*-infected AGS cells. **c**. Efficacy of *G.acid*, *S.thermophilus*, and *L.casei*’s postbiotics on *IL-6* expression in *H.pylori*-infected AGS cells. **d**. Synergistic effects of different combinations of *G.acid*, *S.thermophilus*, and *L.casei*’s postbiotics on *IL-6* expression in *H.pylori*-infected AGS cells. **e**. The activities of *G.acid*, *S.thermophilus*, and *L.casei*’s postbiotics on *IL-1beta* expression in *H.pylori*-infected AGS cells. **f**. Synergistic effects of different combinations of *G.acid*, *S. thermophilus*, and *L.casei*’s postbiotics on *IL-1B* expression in *H.pylori*-infected AGS cells. **g**. Efficacies of *G.acid*, *S.thermophilus*, and *L.casei*’s postbiotics on *IL-8* synthesis in *H.pylori*-infected AGS cells. **h**. Synergistic effects of different combinations of *G.acid, S.thermophilus,* and *L.casei*’s postbiotics on *IL-8* synthesis on *H.pylori* infected AGS cells. **i**. Effects of *G.acid, S.thermophilus*, and *L.casei*’s postbiotics on *IL-10* synthesis in *H.pylori*-infected AGS cells. **j**. Synergistic effects of different combinations of *G.acid, S.thermophilus*, and *L.casei*’s postbiotics on *IL-10* expression in *H.pylori*-infected AGS cells. **k**. Comparison of the effects of *G.acid*, *S.thermophilus,* and *L.casei*’s postbiotics on *COX-2* synthesis with antibiotic treatment in *H.pylori*-infected AGS cells. **l**. Comparison of the synergistic effect of different combinations of *G.acid*, *S.thermophilus*, and *L.casei*’s postbiotics with antibiotic treatment on the *COX-2* gene expression in *H.pylori*-infected AGS cells. **m**. Effects of *G.acid*, *S. thermophilus*, and *L.casei*’s postbiotics on *FOX-M1* expression in *H.pylori*-infected AGS cells. **n**. Synergistic effects of different combinations of *G.acid*, *S.thermophilus*, and *L.casei*’s postbiotics on *FOX-M1* expression in *H.pylori*-infected AGS cells. **o**. Effects of *G. acid, S.thermophilus,* and *L.casei*’s postbiotics on *IL-33* expression in *H.pylori*-infected AGS cells. **p**. Synergistic effects of different combinations of *G.acid, S.thermophilus*, and *L.casei*’s postbiotics on *IL-33* expression in *H.pylori*-infected AGS cells. **r**. Comparison of the effect of *G.acid*, *S.thermophilus*, and *L.casei*’s postbiotics on *NF-kB* expression level in the *H.pylori*-infected AGS cells with the antibiotic group. **s**. Comparison of the synergistic effect of different combinations of *G.acid*, *S.thermophilus*, and *L.casei*’s postbiotics with the antibiotic group on the level of *NF-kB* expression in *H.pylori*-infected AGS cells. **t**. Comparison of gastric *H. pylori* bacterial load among treatment groups based on CFU counts (log₁₀ CFU/g): Demonstrating the impact of postbiotics, *Glycyrrhetinic acid*, and antibiotics

Vero cell experiments identified safe, non-toxic concentrations: *G.acid* (≤ 1.25 µg/mL) and postbiotics (≤ 12.5 µg/mL) (Fig. 1a-c). TNF-α expression remained unchanged in antibiotic and *G.acid* groups but was reduced 10-fold with *S. thermophilus* and 5-fold with *L. casei* postbiotics (Fig. 2a).

The combination of *S. thermophilus* and *L. casei* postbiotics significantly inhibited TNF-α expression. A strong inhibitory effect was also observed in the *G.acid* plus antibiotic group (Fig. 2b).

IL-6 expression in *H. pylori*-infected AGS cells was reduced 10-fold with *S. thermophilus* and *L. casei* postbiotics and 5-fold with *G.acid*, while the antibiotic group showed no significant decrease (Fig. 2c).

Both individual and combined postbiotic treatments significantly reduced IL-6 gene expression. Notably, combinations including *S. thermophilus* with *G. acid*, *L. casei*, or both led to a 10-fold decrease compared to the control. Other combinations also showed significant reductions, while the antibiotic group showed no notable change (Fig. 2d).

*S. thermophilus* and *L. casei* postbiotics showed strong inhibitory effects, reducing gene expression by 10-fold and 5-fold, respectively, compared to the control (Fig. 2e).

Binary, triple, and quaternary combinations of *S. thermophilus*, *L. casei* postbiotics, *G. acid*, and antibiotics significantly reduced IL-1β expression-up to 10-fold-compared to the control, indicating strong synergistic effects (Fig. 2f).

*S. thermophilus* and *L. casei* postbiotics reduced IL-8 expression by 10-fold and 2-fold, respectively, while *G. acid* had no effect. Antibiotic treatment increased IL-8 expression by 2.6-fold (Fig. 2g).

*S. thermophilus* + *L. casei* postbiotics reduced IL-8 expression by 2-fold, while other combinations increased it. Notably, combining both postbiotics with antibiotics led to a 7.1-fold increase vs. control (Fig. 2h).

IL-10 expression did not change significantly with antibiotics or *L. casei* postbiotics but increased 2-fold with *G. acid* and 3.1-fold with *S. thermophilus* postbiotics (Fig. 2i).

Double, triple, and quadruple postbiotic combinations significantly increased IL-10 expression. Notably, *G. acid* + *L. casei* (3.1-fold) and the full combination with antibiotics (3.2-fold) showed the highest increases vs. control (Fig. 2j).

COX-2 expression decreased 10-fold with *S. thermophilus* and 1.67-fold with *L. casei* postbiotics, while antibiotics and *G. acid* showed no significant change vs. control (Fig. 2k).

All combined postbiotic groups showed significantly reduced COX-2 expression vs. control, except *S. thermophilus* + *G. acid*, which showed no change. The antibiotic group showed a 1.6-fold increase. Five-fold reductions were observed in combinations including *L. casei* postbiotics (Fig. 2l).

*FOX-M1* expression decreased significantly with *S. thermophilus* (2.5-fold) and *L. casei* (10-fold) postbiotics, while non-significant increases were seen with antibiotics and *G. acid* (Fig. 2m).

*S. thermophilus* + *G. acid* and *S. thermophilus* + *G. acid* + *L. casei* postbiotics significantly reduced *FOX-M1* expression by 3.3- and 2.5-fold, respectively, while all other groups, including the standard drug, showed increased expression (Fig. 2n).

IL-33 expression significantly decreased in *S. thermophilus* (5-fold) and *G. acid* (10-fold) groups, while the antibiotic group showed no change vs. control (Fig. 2o).

Combined treatments significantly reduced IL-33 expression: 3.3-fold with *S. thermophilus* + *G. acid*, and 5-fold with both *S. thermophilus* + *L. casei* and the triple combination (Fig. 2p).

*S. thermophilus* and *L. casei* postbiotics reduced NF-κB-1 expression 3.3-fold, similar to the antibiotic group, while *G. acid* led to a 2-fold decrease vs. control (Fig. 2r).

Combined postbiotics significantly inhibited NF-κB expression: *S. thermophilus* + *L. casei* showed a 10-fold, and *G. acid* + *L. casei* a 5-fold reduction vs. control, indicating strong synergistic effects (Fig. 2s).

*H. pylori* CFU levels in gastric tissue were quantitatively assessed by culture and reported as log₁₀ CFU/g. The highest bacterial burden was observed in the infected control group (6.14 ± 0.18 log₁₀ CFU/g), whereas the *S. thermophilus* + *L. casei + G. acid* + antibiotic (quaternary) group achieved complete eradication with undetectable CFUs (0.00 ± 0.00 log₁₀ CFU/g). The ternary combination group (*S. thermophilus* + *L. casei* + antibiotic) showed a mean bacterial load of 1.63 ± 0.09 log₁₀ CFU/g. In contrast, binary combinations exhibited varying levels: *S. thermophilus* + antibiotic group 2.83 ± 0.17 log₁₀ CFU/g, *L. casei* + antibiotic group 3.02 ± 0.15 log₁₀ CFU/g, and *G. acid* + antibiotic group 3.24 ± 0.17 log₁₀ CFU/g. All treatment groups demonstrated statistically significant reductions in bacterial load compared to the infected control (*p* < 0.01, chi-square test; Fig. 2t). Furthermore, infection rates among groups were statistically analyzed using the chi-square (χ²) test (*n* = 5 animals per group), and statistical significance was determined by comparing the number of infected and uninfected animals across different treatment groups.

### Animal experiments and histopathological results

After *H. pylori* infection, gastric tissues were collected post-treatment and analyzed via histopathology and culture to assess the presence of *H. pylori*. All rats in the infection control group had *H. pylori*, with severe lymphocyte/leukocyte infiltration and marked mucosal-submucosal edema. In contrast, the *S. thermophilus* postbiotics group showed no immune cell infiltration and only mild submucosal edema, with negative culture results and no *H. pylori*-related pathology.

*H. pylori* was detected in 40% of rats in both the *G. acid* and *L. casei* groups. *G. acid* caused moderate lymphocyte and low leukocyte infiltration, while *L. casei* led to mild lymphocyte infiltration without edema. The antibiotic group had a 20% infection rate with only lymphocyte infiltration.

In the *S. thermophilus* + *G. acid* group, *H. pylori* was detected in 20% of rats, with lymphocyte infiltration but no edema. In contrast, the *L. casei* + *G. acid* group showed 60% infection, along with mucosal edema and immune cell infiltration. The *G. acid* + antibiotic group had 20% infection and lymphocyte infiltration. Triple treatment groups showed better protection and clearer histopathological improvement.

*H. pylori* was not detected in rats treated with *S. thermophilus* + *L. casei* + antibiotics or *G. acid*. Both groups showed moderate lymphocyte infiltration; the latter also showed mild edema and leukocyte infiltration.

Quadruple treatment groups also achieved complete *H. pylori* eradication. No edema or leukocyte infiltration was observed, with only mild lymphocyte infiltration present.

**Fig. 3:**
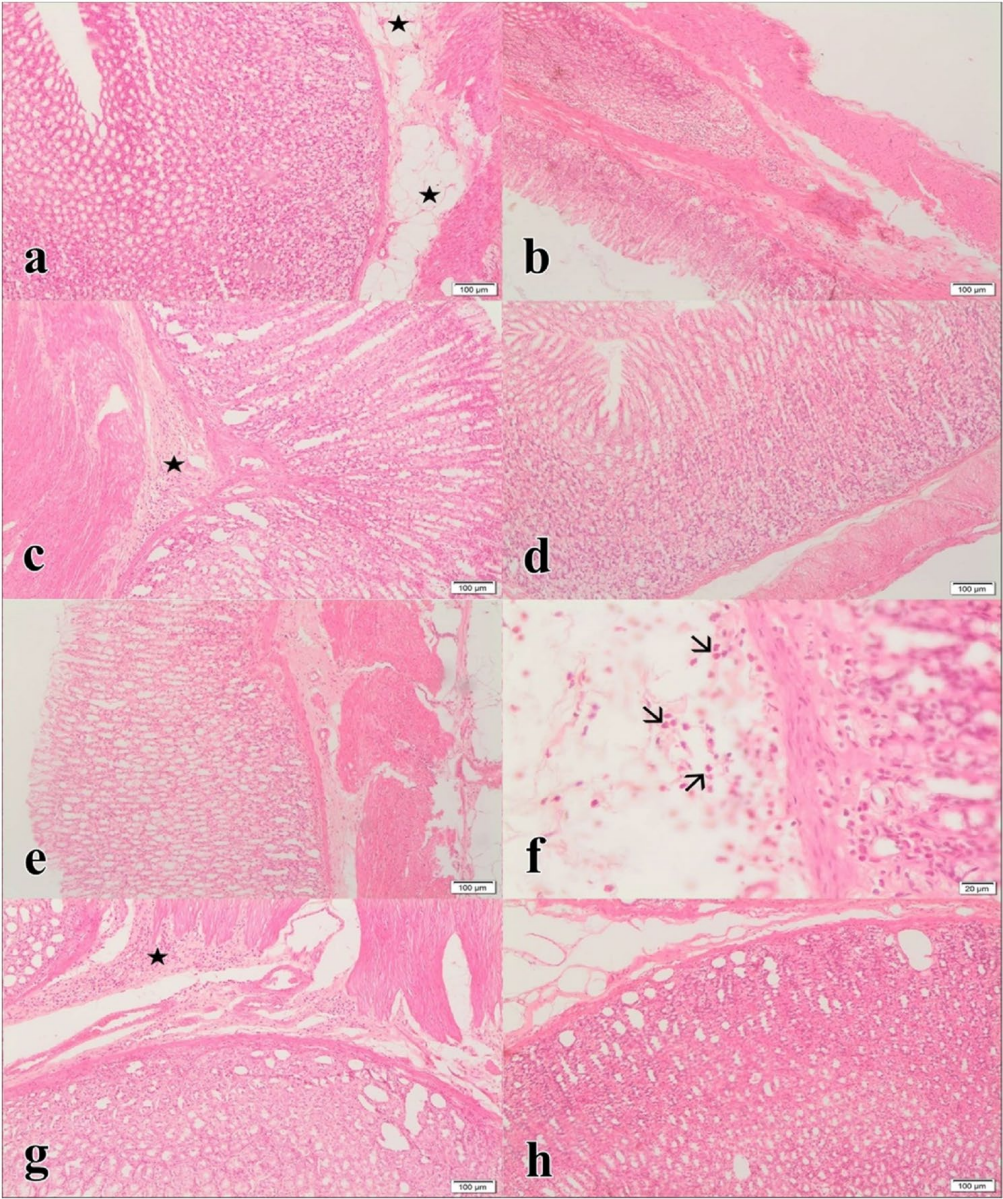
**a***S.thermophilus*’ postbiotics treatment group: Mild edema of the submucosa (stars), H×E. **b***G.acid* treatment group: Lymphoplasmocytic infiltration in the mucosa and submucosa, moderately severe infection, H×E. **c***L.casei*’s postbiotics treatment group: Mild lymphoplasmacytic infiltration in the submucosal region (star), H×E. **d** Antibiotic treatment group, H×E. **e***S.thermophilus*’ postbiotics plus *G.acid* treatment group H×E. **f***S.thermophilus*’ postbiotics plus *Lactobacillus casei*’s postbiotics treatment group: Mild eosinophil leukocyte infiltration in the mucosal and submucosal region (arrows), H×E. **g***G. acid* plus *L.casei*’s postbiotics treatment group: Mild lymphoplasmacytic infiltration in the submucosa (star, star), H×E. **h) ***G.acid* plus antibiotic treatment group, H×E

Samples from the S. thermophilus postbiotics group showed lymphoplasmacytic infiltration in the mucosal and submucosal layers, mild submucosal edema, and no eosinophil leukocyte infiltration (Fig. 3a).

Samples from the *G. acid* treatment group showed moderate mucosal and submucosal lymphoplasmacytic infiltration, mild eosinophil leukocyte infiltration, and no submucosal edema (Fig. 3b).

In the *L. casei* postbiotics treatment group, mild lymphoplasmacytic infiltration was observed in the mucosal and submucosal regions, with no eosinophil infiltration or submucosal edema (Fig. 3c).

In the antibiotic treatment group, mild lymphoplasmacytic infiltration was observed in the mucosal and submucosal regions, with no eosinophil infiltration or submucosal edema (Fig. 3d).

No mucosal or submucosal lymphoplasmacytic infiltration, eosinophil infiltration, or edema was observed in the *S. thermophilus* postbiotics + *G. acid* treatment group (Fig. 3e).

The *S. thermophilus* + *L. casei* group showed mild lymphoplasmacytic and eosinophil infiltration with submucosal edema (Fig. 3f).

In the *G. acid* + *L. casei* postbiotics group, mild lymphoplasmacytic infiltration was observed in the mucosal and submucosal regions, with no eosinophil infiltration or submucosal edema (Fig. 3g).

In the *G. acid* + antibiotic group, mild mucosal and submucosal lymphoplasmacytic infiltration was observed, with no eosinophil infiltration or submucosal edema (Fig. 3h).

**Fig. 4:**
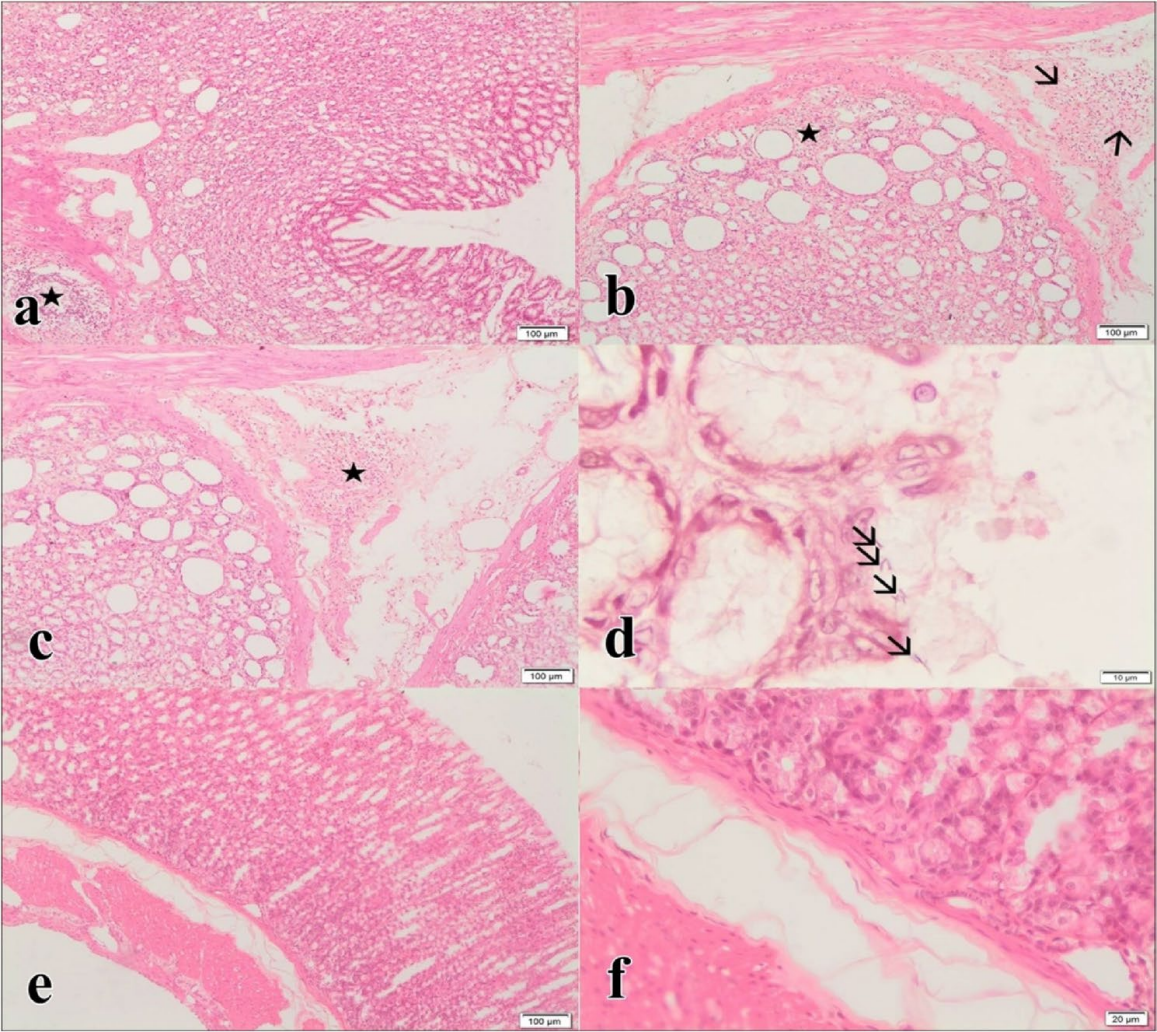
**a***S.thermophilus’* postbiotics plus *L.casei*’s postbiotics plus antibiotic treatment group: Moderate lymphoplasmacytic infiltration in the mucosal and submucosal region (star), H×E. **b***S.thermophilus*’ postbiotics plus *G.acid* plus *L.casei*’s postbiotics treatment group: Moderate lymphoplasmacytic infiltration in the mucosa (star) and submucosal region (arrows), H×E. **c) ***G.acid* plus *L.casei*’s postbiotics plus *S.thermophilus*’ postbiotics plus Antibiotic treatment group: Moderate lymphoplasmacytic infiltration in the mucosal and submucosal region (star), H×E. **d** Infection control group. *H.pylori* colonization (arrows), 100×, H×E.**e** Negative control group, H×E. **f)** Negative control group, H×E

In the *S. thermophilus* + *L. casei* + antibiotic group, moderate lymphoplasmacytic infiltration was observed in the mucosal and submucosal regions, with no eosinophil infiltration or edema (Fig. 4a).

In the *S. thermophilus* + *L. casei* + *G. acid* group, moderate lymphoplasmacytic infiltration was observed in the mucosal and submucosal regions, along with mild eosinophil infiltration and submucosal edema (Fig. 4b).

In the *S. thermophilus* + *L. casei* + *G. acid* group, moderate lymphoplasmacytic infiltration, mild eosinophil infiltration, and submucosal edema were observed (Fig. 4c).

In the infection control group, *H. pylori* colonization was detected with severe lymphocytic infiltration, mild edema, and eosinophil infiltration in the mucosal and submucosal regions (Fig. 4d).

In the negative control group (non-infected), no lymphoplasmacytic infiltration, eosinophil infiltration, or edema was observed in the mucosal and submucosal regions (Fig. 4e and f).

## Discussion

Recently, combating drug resistance has focused on preventing antibiotic resistance and enhancing synergistic effects through compound combinations. Natural products, especially postbiotics-bioactive metabolites from probiotics, are widely used for their antimicrobial properties. Their potential to create synergistic effects offers a promising strategy against drug resistance [43–48].

Synergistic effects, where combined compounds exhibit more potent activity than individually, are promising alternatives amid rising antibiotic resistance and treatment challenges [49, 50].

Increasing antimicrobial resistance has boosted interest in natural bioactive compounds, which enhance antimicrobial effectiveness either directly or indirectly by modifying antibiotic resistance [7, 51]. Combinations of natural compounds may alter or block microbial resistance mechanisms, reducing or eliminating antibiotic resistance, thus representing a promising approach [52].

*H. pylori* infection promotes gastric inflammation by increasing proinflammatory cytokines (TNF-α, IL-1β, IL-6), favoring carcinogenesis. Thus, controlling or inhibiting these cytokines is crucial during treatment [53].

*S. thermophilus* and *L. casei* postbiotics individually inhibited TNF-α synthesis, while *G. acid* and antibiotics alone had no effect. Significant synergistic inhibition occurred only in the *G. acid* + antibiotic and quadruple (*S. thermophilus* + *L. casei* + *G. acid* + antibiotic) groups, suggesting potential improvement of conventional therapy.

IL-6 is rapidly produced during infections or injuries, but uncontrolled, prolonged IL-6 synthesis may lead to chronic inflammation and autoimmune pathology [54].

In this study, *S. thermophilus*, *L. casei* postbiotics, and *G. acid* significantly reduced IL-6 synthesis compared to control, indicating controlled inflammation, while antibiotics alone had no effect. Strong reductions occurred with combinations of *S. thermophilus* + *G. acid*, *S. thermophilus* + *L. casei*, and their triple combination. Quadruple combinations including antibiotics further reduced IL-6 expression, suggesting enhanced antibiotic efficacy against *H. pylori*. Controlling IL-1β, a key inflammatory mediator involved in carcinogenesis, is also crucial.

Elevated IL-1β is linked to cancer progression [55]. In our study, IL-1β expression significantly decreased with *S. thermophilus* and *L. casei* postbiotics, while *G. acid* and antibiotics alone showed no significant effect. Combined treatments showed greater efficacy.

The combined *S. thermophilus* + *L. casei* postbiotics and the quadruple combination (*S. thermophilus* + *L. casei* + *G. acid* + antibiotics) showed strong efficacy. *G. acid* alone did not significantly reduce IL-1β, but its combination with *L. casei* postbiotics and antibiotics exhibited notable synergistic activity.

IL-8 is overexpressed in *H. pylori*-infected gastric mucosa and associated with poor gastric cancer prognosis. It has chemotactic properties and promotes cancer cell proliferation and migration [56, 57].

The *L.casei*’s postbiotics and *S.thermophilus*’s postbiotics caused inhibition of *IL-8* expression, which was significant in the *S.thermophilus*’s postbiotics treatment group compared to the control. While there was no increase in *IL-8* expression in the *G. acid* treatment group, *IL-8* expression was significantly higher in the antibiotic treatment group than in the control group. This situation can be interpreted as the combined antibiotic therapy used in the treatment of *H.pylori* today may pose a threat to cancer progression by increasing *IL-8* expression. One of the most substantial findings in the study was the significant inhibition of *IL-8* synthesis in the quadruple combined treatment group (*S.thermophilus*’s postbiotics plus *L.casei*’s postbiotics plus *G.acid* plus antibiotics), as with other cytokines, which was considered very important in creating new treatment protocols.

Alleviating the high inflammatory response in *H.pylori* infection is directly related to patient well-being and treatment success. Interleukin 10 (*IL-10*) is an anti-inflammatory cytokine involved in the down-regulation of human immune reactions. *IL-8* is effective in reducing the increased inflammatory response in *H.pylori* infection [58]. We found a significant increase in *IL-10* synthesis in the *L.casei* and antibiotic treatment group compared to the control group. There was a significant increase in *IL-10* synthesis in the *G.acid* and especially *S.thermophilus*’s postbiotics treatment group. Interestingly, there were significant increases in *IL-10* expression in all treatment groups with double, triple, and quadruple combinations of compounds compared to the standard antibiotic treatment group. Strong synergistic activity was found in the increase of *IL-10* expression, especially in the quadruple combined treatment group. These findings were considered extremely important in the improvement of new treatment approaches or existing conventional therapy modalities.

COX-2, involved in converting arachidonic acid to prostaglandins, contributes to *H. pylori*-associated gastric carcinogenesis and is often overexpressed in gastric cancers. Thus, inhibiting COX-2 synthesis is crucial for preventing cancer progression [59].

Antibiotic therapy significantly increased COX-2 expression, potentially predisposing to cancer progression. *S. thermophilus* postbiotics significantly reduced COX-2 expression, whereas *L. casei* postbiotics caused a non-significant decrease. All combined treatments, except *S. thermophilus* + *G. acid*, significantly inhibited COX-2. Thus, combining these compounds with antibiotics may substantially reduce gastric cancer risk.

FOX-M1, a transcription factor commonly overexpressed in tumors, promotes cancer cell proliferation, migration, and angiogenesis. Thus, inhibiting FOX-M1 synthesis is crucial for preventing cancer progression [60, 61].

Postbiotics from *S. thermophilus* and *L. casei* significantly inhibited FOX-M1 expression (Fig. 2m). Combined treatments of *S. thermophilus* + *G. acid* and *S. thermophilus* + *L. casei* + *G. acid* also notably suppressed FOX-M1 (Fig. 2n).

Increased levels of *IL-33* expression in the gastric mucosa of patients infected with *H.pylori* are considered a signal of the protumorigenic effect of this cytokine. In the present study, a decrease in *IL-33* expression was observed in all groups compared to the control group. The decrease was significant in the *S.thermophilus* and *L.casei*’s postbiotics, and *G. acid* treatment groups. However, there was no significant decrease in the antibiotic treatment group, which was considered active inflammation. We believe this may be due to the lower antibacterial activity than other groups. There was no significant reduction in *IL-33* expression in the groups treated with a combination of compounds and antibiotics. The absence of an increase in *IL-33* expression in the treatment groups was considered the end of the acute phase of the disease and the transition to the recovery phase.

NF-κB, a proinflammatory pathway activated by *H. pylori* cagA during gastric carcinogenesis, promotes cytokine release, proliferation, angiogenesis, invasion, and apoptosis inhibition [62]. Chemotherapy-induced cellular stress activates NF-κB, potentially causing drug resistance. NF-κB is also a key factor in inflammation-driven carcinogenesis, including gastrointestinal cancers [63–65].

Postbiotics may exert their effects by regulating the NF-κB pathway. Mechanistically, postbiotic metabolites such as butyrate and lactate have been shown to inhibit NF-κB translocation by modulating IκB kinase activity or acting on upstream pattern recognition receptors such as TLRs. Relevant literature supports their influence on signal transduction pathways leading to suppressed cytokine expression [66–68]. To better explain the observed differences in biological activity between *S. thermophilus* and *L. casei* postbiotics, it is essential to consider their distinct compositional profiles. *S. thermophilus*-derived postbiotics predominantly contain exopolysaccharides, short-chain fatty acids, and specific bacteriocins, which have been associated with immunomodulatory and anti-inflammatory properties [69, 70]. In contrast, *L. casei*-derived postbiotics are rich in peptidoglycan fragments, lipoteichoic acids, and lactic acid, which play crucial roles in regulating cytokines and enhancing mucosal immunity [71, 72]. These compositional distinctions may explain the observed variations in their individual and combined immunomodulatory and anti-inflammatory effects observed in this study.

Therefore, inhibiting this gene transcription during *H. pylori* infection is of great importance in preventing cancer progression. *NF-κB* expression was inhibited in the single and combined treatment groups. Vigorous synergistic activity was detected in the *S. thermophilus* postbiotics plus *L. casei* postbiotics combined treatment group. We believe that the therapeutic activities of these compounds and combinations are very substantial in inhibiting *NF-κB* gene expression, both to prevent drug resistance and to inhibit cancer progression.

In addition to gene expression results, microbial load analysis revealed a significant reduction in *H. pylori* colonization across all treatment groups. Notably, the quadruple combination group exhibited complete eradication (0.00 ± 0.00 log10 CFU/g), further supporting the therapeutic potential of compound synergy.

After sacrifice, gastric tissues were analyzed microbiologically and histopathologically. Among single-treatment groups, *S. thermophilus* postbiotics showed complete *H. pylori* eradication and significant histopathological improvement, characterized by absence of lymphocyte infiltration, low leukocyte infiltration, and mild edema, indicating full recovery.

In the group treated with postbiotics of *S.thermophilus*, there was a noticeable improvement in both microbiological and histological conditions. This improvement is believed to be due to the regulatory effect of *S.thermophilus* postbiotics on gene expressions related to inflammation, specifically *NF-kB*. The expression levels of *TNF-alpha*, *COX-2*,* IL-1 beta*,* IL-6*,* IL-33*,* FOX-M1*, and *IL-8* were significantly reduced in the *S.thermophilus* postbiotics group, while the *IL-10* expression was significantly increased. We believe the effect is crucial for histopathological recovery, and recovery depends on gene expressions and their inhibition. Notably, this curative effect was not observed in the groups treated with single *G.acid*, *L.casei*’s postbiotics, or conventional antibiotics.

In the *S. thermophilus* postbiotics group, *H. pylori* was eradicated, unlike other single-treatment groups (*G. acid*: 40%, *L. casei*: 40%, antibiotics: 20% isolation rates). Culture findings matched histopathology: lymphoplasmacytic infiltration persisted in the antibiotic group, accompanied by leukocyte infiltration and edema in the *L. casei* group, and moderate lymphoplasmacytic with mild leukocyte infiltration in the *G. acid* group.

In combined groups (*S. thermophilus* + *G. acid*, *S. thermophilus* + *L. casei*, *G. acid* + antibiotic), *H. pylori* eradication was incomplete, but histological recovery was notable. The *S. thermophilus* + *G. acid* group showed no edema, leukocyte, or lymphocyte infiltration. The absence of edema and leukocyte infiltration in the *G. acid* + antibiotic group suggests enhanced antibiotic efficacy.

Triple combination therapies (*S. thermophilus* + *L. casei* + antibiotic and *S. thermophilus* + *G. acid* + antibiotic) were more effective against *H. pylori*, achieving complete eradication and notable histological improvement. Only moderate lymphocyte infiltration, mild edema, and minor leukocyte infiltration were observed.

Among tested regimens, quaternary combinations (four-component treatments; *S. thermophilus + L. casei* + *G. acid* + antibiotics) yielded complete eradication with undetectable CFUs (0.00 ± 0.00 log₁₀ CFU/g) and the most comprehensive histological recovery. Ternary combinations (three-component treatments; *S. thermophilus* + *L. casei* + antibiotics) showed significant but partial reductions in bacterial load (1.63 ± 0.09 log₁₀ CFU/g), indicating an intermediate level of efficacy. Binary combinations (two-component treatments: *S. thermophilus* + antibiotics, *L. casei* + antibiotics, or *G. acid* + antibiotics) exhibited variable effects, with CFU levels ranging between 2.8 and 3.1 log₁₀ CFU/g, reflecting modest reductions. This gradient in effectiveness underscores the synergistic value of multi-component therapies against *H. pylori*, highlighting their potential integration into future therapeutic protocols.

In the quadruple treatment (*S. thermophilus* + *L. casei* + *G. acid* + antibiotic), *H. pylori* was entirely eradicated, with no leukocyte infiltration or edema and only mild lymphocyte infiltration. Complete eradication and significant histological recovery were also observed in the single *S. thermophilus* group.

Eradicating *H. pylori*, a critical risk factor for gastric cancer is essential for cancer prevention. Our study presents a promising new treatment that effectively eliminates this bacterium, potentially preventing gastric cancer development and significantly impacting patient outcomes.

Compared to previous studies on probiotics [13, 33], the postbiotics utilized in this study offer several distinct advantages. While probiotics require live bacterial viability and successful colonization in the gastrointestinal tract to exert their beneficial effects, postbiotics function through their bioactive metabolites, such as short-chain fatty acids, bacteriocins, and polysaccharides, eliminating the dependency on live organisms. This not only enhances safety, especially in immunocompromised hosts, but also ensures greater stability, shelf-life, and batch-to-batch consistency. Moreover, unlike probiotics, whose colonization efficiency may be affected by host microbiota and antibiotic use, postbiotics provide a predictable and controlled biological response, as demonstrated by the consistent anti-inflammatory and anti-*Helicobacter pylori* effects were observed in both in vitro and in vivo models in this study. These attributes position postbiotics as promising next-generation therapeutic agents for managing *H. pylori* infection.

Quantitative culture-based CFU analysis strongly aligned with histological improvement. Groups receiving *S. thermophilus* postbiotics alone or in combination achieved significantly lower bacterial burdens, with full eradication in the quadruple group (*S. thermophilu*s + *L. casei* + *G. acid* + antibiotic), indicating that bacterial load can serve as a reliable indicator of treatment efficacy.

*G.acid* combined with *S.thermophilus* and *L.casei*’s postbiotics, *G.acid* exhibited remarkable effectiveness against *H.pylori* in the AGS cell line and experimental rat gastritis model. The molecular mechanism underlying this efficacy involves the suppression of pro-inflammatory cytokines and the induction of anti-inflammatory cytokines, leading to the reduction of gastric inflammation and the prevention of *H.pylori* colonization. The findings of both in-vitro and in-vivo experiments have significant results for the obliteration of *H.pylori*. These results can cause the eradication of *H.pylori* and may be a breakthrough for its treatment, one of the most common infectious agents in the world. The success in treating *H.pylori*, as well as the treatment and prevention of H.pylori-related inflammatory disorders and gastric cancer, may be influential. These findings may have crucial consequences for exploring new treatment methods against *H.pylori* infections and improving existing treatment methods. However, it should be noted that a limitation of this study was the absence of cytokine protein level measurements in vivo, due to technical limitations in consistently isolating high-quality RNA or cytokine proteins from inflamed gastric tissue. 

## Supplementary Information

Supplementary Material 1.

## Data Availability

All data generated or analyzed during this study are included in this published article and its supplementary information files.
