# Supplementary material for: The effects of postbiotics and glycyrrhetinic acid on immune response and inflammation-related genes during H. pylori eradication therapy
Source: BMC Microbiol. 2025 Dec 13;26:39. doi: 10.1186/s12866-025-04191-1 (PMC12821235; doi:10.1186/s12866-025-04191-1)
Supplement: Supplementary file 1 — Supplementary Material 1. [file 12866_2025_4191_MOESM1_ESM.docx]

**Tables**

**Table 1.** Experimental groups and numbers in gene expression study.

| Groups | The number of repetitions |
| --- | --- |
| Group 1 (HP group) | 3 |
| Group 2 (STP group) | 3 |
| Group 3 (GA group) | 3 |
| Group 4 (LCP group) | 3 |
| Group 5 (AB group | 3 |
| Group 6 (STP+GA group) | 3 |
| Group 7 (STP+LCP group) | 3 |
| Group 8 (GA+LCP group) | 3 |
| Group 9 (GA+AB group) | 3 |
| Group 10 (STP+LCP+AB group) | 3 |
| Group 11 (STP+GA+LCP group) | 3 |
| Group 12 (STP+GA+LCP+AB group) | 3 |

*HP: H.pylori*

*STP: S.thermophilus’ postbiotics*

*GA: Glysithetinic acid*

*LCP: L.casei’s postbiotics*

*AB: (Amoxicillin+Clarithromycin)*

**Table 2.** Oligonucleotide sequences were used in the present study.

| Primers | Sequences | References |
| --- | --- | --- |
| GAPDH | 5‘-TGCACCACCAACTGCTTAGC-3‘ | 31 |
|  | 5‘-GGCATGGACTGTGGTCATGAG-3’ |  |
| IL-33 | 5’-GGAAGAACACAGCAAGCAAAGCCT-3’ | 32 |
|  | 5’-TAAGGCCAGAGCGGAGCTTCATAA-3’ |  |
| FOX-M1 | 5’-TGCAGCTAGGGATGTGAATCTTC-3’ | 33 |
|  | 5’-GGAGCCCAGTCCATCAGAACT-3’ |  |
| COX-2 | 5′-CTTGCTGTTCCCACCCATGTCAAA-3′ | 34 |
|  | 5′-TGCACTGTGTTTGGAGTGGGTTTC-3′ |  |
| TNF-alpha | 5′-CGAGTGACAAGCCTGTAGC-3′ | 35 |
|  | 5′-GGTGTGGGTGAGGAGCACAT-3′ |  |
| IL-10 | 5′-GTGATGCCCCAAGCTGAGA-3′ | 36 |
|  | 5′-CACGGCCTTGCTCTTGTTTT-3′ |  |
| IL-6 | 5′-AAATGCCAGCCTGCTGA CGAAC-3′ | 34 |
|  | 5′-AACAACAATCTGAGGTGCCCATGCTAC-3′ |  |
| IL-1 beta | 5’-AAGCCCTTGCTGTAGTGGTG-3’ | 37 |
|  | 5’-GAAGCTGATGGCCCTAAACA-3’ |  |
| IL-8 | 5’-AGCACTCCTTGGCAAAACTG-3’ | 38 |
|  | 5’-CGGAAGGAACCATCTCACTG-3’ |  |
| Nf-kB | 5’-AAAGACACATCCGGACCTCG-3’ | 39 |
|  | 5’-TGTAAGAGTTCCCCTCCGGT-3’ |  |

**Table 3.** Animal experiments treatment groups and numbers.

| Experimental groups | Numbers of rats |
| --- | --- |
| Group 1 (HP group) | 5 |
| Group 2 (Healthy control group) | 5 |
| Group 3 (STP treatment group) | 5 |
| Group 4 (GA treatment group) | 5 |
| Group 5 (LCP treatment group) | 5 |
| Group 6 (AB group) | 5 |
| Group 7 (STP+GA group) | 5 |
| Group 8 (STP+LCP group) | 5 |
| Group 9 (GA+LCP group) | 5 |
| Group 10 (GA+AB group) | 5 |
| Group 11 (STP+LCP+AB group) | 5 |
| Group 12 (STP+GA+LCP group) | 5 |
| Group 13 (STP+GA+LCP+AB group) | 5 |
| Total number of animals | 65 |

*HP*: *H.pylori*

*STP*: *S.thermophilus*’ postbiotics

*GA*: *Glysithetinic acid*

*LCP*: *L.casei*’s postbiotics

*AB*: *(Amoxicillin+Clarithromycin)*

**Figures**

| Groups | Summary | P value | Groups | Summary | P value |
| --- | --- | --- | --- | --- | --- |
| Control vs. 2.5 µg/ml | ns | 0,9991 | Control vs. 12.5 µg/ml | ns | 0,6877 |
| Control vs. 5.0 µg/ml | ns | 0,5603 | Control vs. 15.0 µg/ml | * | 0,0112 |
| Control vs. 7.5 µg/ml | ns | 0,9849 | Control vs. 17.5 µg/ml | *** | 0,0002 |
| Control vs. 10.0 µg/ml | ns | 0,9071 | Control vs. 20.0 µg/ml | **** | <0,0001 |

| Groups | Summary | P value | Groups | Summary | P value |
| --- | --- | --- | --- | --- | --- |
| Control vs. 2.5 µg/ml | ns | 0,9996 | Control vs. 12.5 µg/ml | ns | 0,7794 |
| Control vs. 5.0 µg/ml | ns | 0,9864 | Control vs. 15.0 µg/ml | ** | 0,0024 |
| Control vs. 7.5 µg/ml | ns | 0,8085 | Control vs. 17.5 µg/ml | **** | <0,0001 |
| Control vs. 10.0 µg/ml | ns | 0,9864 | Control vs. 20.0 µg/ml | **** | <0,0001 |

| Groups | Summary | P value | Groups | Summary | P value |
| --- | --- | --- | --- | --- | --- |
| Control vs. 0.25 µg/ml | ns | 0,7520 | Control vs. 1.25 µg/ml | ns | 0,8611 |
| Control vs. 0.50 µg/ml | ns | 0,9989 | Control vs. 1.50 µg/ml | ** | 0,0032 |
| Control vs. 0.75 µg/ml | ns | 0,8279 | Control vs. 1.75 µg/ml | **** | <0,0001 |
| Control vs. 1.0 µg/ml | ns | 0,5788 | Control vs. 2.00 µg/ml | **** | <0,0001 |

**Figures 1a-c.** Cytotoxicity results of the *S.thermophilus*' (a) and *L.casei*’s (b) postbiotics, and *G.acid* (c) on the viability of Vero cells by MTT method.

**Figure 2a.** Effects of *G. acid*, *S. thermophilus,* and *L.casei*’s postbiotics on TNF-alpha expression on *H.pylori*-infected AGS cells.

| TNF-alpha | Summary | P value | TNF-alpha | Summary | P value |
| --- | --- | --- | --- | --- | --- |
| Control vs. STP | **** | <0,0001 | STP vs. LCP | ns | 0,1922 |
| Control vs. GA | ** | 0,0013 | STP vs. AB | **** | <0,0001 |
| Control vs. LCP | **** | <0,0001 | GA vs. LCP | **** | <0,0001 |
| Control vs. AB | * | 0,0187 | GA vs. AB | ns | 0,3971 |
| STP vs. GA | **** | <0,0001 | LCP vs. AB | **** | <0,0001 |

**Figure 2b.** Synergistic effects of different combinations of *G. acid*, *S. thermophilus*, and *L.casei*’s postbiotics with the antibiotic group on TNF-alpha expression in *H.pylori*-infected AGS cells.

| TNF-alpha | Summary | P value | TNF-alpha | Summary | P value |
| --- | --- | --- | --- | --- | --- |
| Control vs. AB | ns | 0,9381 | Control vs. GA+AB | **** | <0,0001 |
| Control vs. STP+GA | * | 0,0376 | Control vs. STP+LCP+AB | **** | <0,0001 |
| Control vs. STP+LCP+AB | **** | <0,0001 | Control vs. STP+GA+LCP+AB | ns | 0,9873 |
| Control vs. GA+LCP | **** | <0,0001 | Control vs. STP+GA+LCP+AB | **** | <0,0001 |

**Figure 2c.** Efficacy of *G. acid*, *S. thermophilus*, and *L.casei*’s postbiotics on IL-6 expression in *H.pylori*-infected AGS cells.

| IL-6 | Summary | P value | IL-6 | Summary | P value |
| --- | --- | --- | --- | --- | --- |
| Control vs. STP | **** | <0,0001 | STP vs. LCP | ns | 0,9994 |
| Control vs. GA | **** | <0,0001 | STP vs. AB | **** | <0,0001 |
| Control vs. LCP | **** | <0,0001 | GA vs. LCP | ns | 0,6523 |
| Control vs. AB | ns | 0,2036 | GA vs. AB | **** | <0,0001 |
| STP vs. GA | ns | 0,7739 | LCP vs. AB | **** | <0,0001 |

**Figure 2d.** Synergistic effects of different combinations of *G. acid*, *S. thermophilus*, and *L.casei*’s postbiotics on IL-6 expression in *H.pylori*-infected AGS cells.

| IL-6 | Summary | P value | IL-6 | Summary | P value |
| --- | --- | --- | --- | --- | --- |
| Control vs. AB | ns | 0,1215 | GA+LCP vs. STP+LCP+AB | ** | 0,0035 |
| Control vs. STP+GA | **** | <0,0001 | GA+LCP vs. STP+GA+LCP+AB | ns | 0,9311 |
| Control vs. STP+LCP+AB | **** | <0,0001 | GA+LCP vs. STP+GA+LCP+AB | ns | >0,9999 |
| Control vs. GA+LCP | **** | <0,0001 | GA+AB vs. STP+LCP+AB | ns | 0,8718 |
| Control vs. GA+AB | **** | <0,0001 | GA+AB vs. STP+GA+LCP+AB | ** | 0,0050 |
| Control vs. STP+LCP+AB | **** | <0,0001 | GA+AB vs. STP+GA+LCP+AB | ns | 0,0716 |
| Control vs. STP+GA+LCP+AB | **** | <0,0001 | STP+LCP+AB vs. STP+GA+LCP+AB | *** | 0,0003 |
| GA+AB vs. STP+GA+LCP+AB | ns | 0,0716 | STP+LCP+AB vs. STP+GA+LCP+AB | ** | 0,0039 |

**Figure 2e.** The activities of *G. acid*, *S. thermophilus*, and *L.casei*’s postbiotics on IL-1 beta expression in *H.pylori*-infected AGS cells.

| IL-1 beta | Summary | P value | IL-1 beta | Summary | P value |
| --- | --- | --- | --- | --- | --- |
| Control vs. STP | **** | <0,0001 | STP vs. LCP | ns | 0,6500 |
| Control vs. GA | *** | 0,0004 | STP vs. AB | ** | 0,0013 |
| Control vs. LCP | **** | <0,0001 | GA vs. LCP | ** | 0,0066 |
| Control vs. AB | *** | 0,0003 | GA vs. AB | ns | 0,9993 |
| STP vs. GA | *** | 0,0010 | LCP vs. AB | ** | 0,0093 |

**Figure 2f.** Synergistic effects of different combinations of *G. acid*, *S. thermophilus*, and *L.casei*’s postbiotics on IL-1B expression in *H.pylori*-infected AGS cells.

| IL-1 beta | Summary | P value | IL-1 beta | Summary | P value |
| --- | --- | --- | --- | --- | --- |
| Control vs. AB | **** | <0,0001 | Control vs. GA+AB | **** | <0,0001 |
| Control vs. STP+GA | **** | <0,0001 | Control vs. STP+LCP+AB | **** | <0,0001 |
| Control vs. STP+LCP+AB | **** | <0,0001 | Control vs. STP+GA+LCP+AB | **** | <0,0001 |
| Control vs. GA+LCP | **** | <0,0001 | Control vs. STP+GA+LCP+AB | **** | <0,0001 |

**Figure 2g.** Efficacies of *G.acid*, *S.thermophilus*, and *L.casei*’s postbiotics on IL-8 synthesis in *H.pylori*-infected AGS cells.

| IL-8 | Summary | P value | IL-8 | Summary | P value |
| --- | --- | --- | --- | --- | --- |
| Control vs. STP | *** | 0,0002 | STP vs. LCP | ns | 0,0532 |
| Control vs. GA | ns | 0,6607 | STP vs. AB | **** | <0,0001 |
| Control vs. LCP | * | 0,0133 | GA vs. LCP | ** | 0,0019 |
| Control vs. AB | **** | <0,0001 | GA vs. AB | **** | <0,0001 |
| STP vs. GA | **** | <0,0001 | LCP vs. AB | **** | <0,0001 |

**Figure 2h.** Synergistic effects of different combinations of *G. acid, S. thermophilus,* and *L.casei*’s postbiotics on IL-8 synthesis on *H.pylori* infected AGS cells.

| IL-8 | Summary | P value | IL-8 | Summary | P value |
| --- | --- | --- | --- | --- | --- |
| Control vs. AB | *** | 0,0002 | Control vs. GA+AB | **** | <0,0001 |
| Control vs. STP+GA | **** | <0,0001 | Control vs. STP+LCP+AB | **** | <0,0001 |
| Control vs. STP+LCP+AB | ns | 0,5140 | Control vs. STP+GA+LCP+AB | **** | <0,0001 |
| Control vs. GA+LCP | * | 0,0107 | Control vs. GA+AB | **** | <0,0001 |

**Figure 2i.** Effects of *G. acid, S. thermophilus*, and *L.casei*’s postbiotics on IL-10 synthesis in *H.pylori*-infected AGS cells.

| IL-10 | Summary | P value | IL-10 | Summary | P value |
| --- | --- | --- | --- | --- | --- |
| Control vs. STP | **** | <0,0001 | STP vs. LCP | **** | <0,0001 |
| Control vs. GA | **** | <0,0001 | STP vs. AB | **** | <0,0001 |
| Control vs. LCP | ns | >0,9999 | GA vs. LCP | **** | <0,0001 |
| Control vs. AB | ns | 0,5866 | GA vs. AB | **** | <0,0001 |
| STP vs. GA | **** | <0,0001 | LCP vs. AB | ns | 0,5866 |

**Figure 2j**. Synergistic effects of different combinations of *G. acid, S.thermophilus*, and *L.casei*’s postbiotics on IL-10 expression in *H.pylori*-infected AGS cells.

| IL-10 | Summary | P value | IL-10 | Summary | P value |
| --- | --- | --- | --- | --- | --- |
| Control vs. AB | ns | >0,9999 | Control vs. GA+AB | * | 0,0251 |
| Control vs. STP+GA | **** | <0,0001 | Control vs. STP+LCP+AB | **** | <0,0001 |
| Control vs. STP+LCP+AB | **** | <0,0001 | Control vs. STP+GA+LCP+AB | *** | 0,0005 |
| Control vs. GA+LCP | **** | <0,0001 | Control vs. STP+GA+LCP+AB | **** | <0,0001 |

**Figure 2k.** Comparison of the effects of GA, *S.thermophilus,* and *L.casei*’s postbiotics on COX-2 synthesis with antibiotic treatment in *H.pylori*-infected AGS cells.

| COX-2 | Summary | P value | COX-2 | Summary | P value |
| --- | --- | --- | --- | --- | --- |
| Control vs. STP | **** | <0,0001 | STP vs. LCP | ** | 0,0028 |
| Control vs. GA | ns | 0,1870 | STP vs. AB | **** | <0,0001 |
| Control vs. LCP | * | 0,0167 | GA vs. LCP | *** | 0,0006 |
| Control vs. AB | *** | 0,0010 | GA vs. AB | * | 0,0328 |
| STP vs. GA | **** | <0,0001 | LCP vs. AB | **** | <0,0001 |

**Figure 2l.** Comparison of the synergistic effect of different combinations of *G. acid*, *S. thermophilus*, and *L.casei*’s postbiotics with antibiotic treatment on the COX-2 gene expression in *H.pylori*-infected AGS cells.

| COX-2 | Summary | P value | COX-2 | Summary | P value |
| --- | --- | --- | --- | --- | --- |
| Control vs. AB | *** | 0,0003 | Control vs. GA+AB | **** | <0,0001 |
| Control vs. STP+GA | ns | 0,9592 | Control vs. STP+LCP+AB | **** | <0,0001 |
| Control vs. STP+LCP+AB | **** | <0,0001 | Control vs. STP+GA+LCP+AB | **** | <0,0001 |
| Control vs. GA+LCP | **** | <0,0001 | Control vs. STP+GA+LCP+AB | **** | <0,0001 |

**Figure 2m.** Effects of *G. acid*, *S. thermophilus*, and *L.casei*’s postbiotics on FOX-M1 expression in *H.pylori*-infected AGS cells.

| FOX-M1 | Summary | P value | FOX-M1 | Summary | P value |
| --- | --- | --- | --- | --- | --- |
| Control vs. STP | **** | <0,0001 | STP vs. LCP | * | 0,0157 |
| Control vs. GA | *** | 0,0001 | STP vs. AB | **** | <0,0001 |
| Control vs. LCP | **** | <0,0001 | GA vs. LCP | **** | <0,0001 |
| Control vs. AB | **** | <0,0001 | GA vs. AB | ** | 0,0061 |
| STP vs. GA | **** | <0,0001 | LCP vs. AB | **** | <0,0001 |

**Figure 2n.** Synergistic effects of different combinations of *G. acid*, *S. thermophilus*, and *L.casei*’s postbiotics on FOX-M1 expression in *H.pylori*-infected AGS cells.

| FOX-M1 | Summary | P value | FOX-M1 | Summary | P value |
| --- | --- | --- | --- | --- | --- |
| Control vs. AB | * | 0,0126 | Control vs. GA+AB | **** | <0,0001 |
| Control vs. STP+GA | * | 0,0286 | Control vs. STP+LCP+AB | **** | <0,0001 |
| Control vs. STP+LCP+AB | *** | 0,0009 | Control vs. STP+GA+LCP+AB | ns | 0,0699 |
| Control vs. GA+LCP | **** | <0,0001 | Control vs. STP+GA+LCP+AB | ** | 0,0033 |

**Figure 2o.** Effects of *G. acid, S. thermophilus,* and *L.casei*’s postbiotics on IL-33 expression in *H.pylori*-infected AGS cells.

| IL-33 | Summary | P value | IL-33 | Summary | P value |
| --- | --- | --- | --- | --- | --- |
| Control vs. STP | **** | <0,0001 | STP vs. LCP | *** | 0,0002 |
| Control vs. GA | **** | <0,0001 | STP vs. AB | **** | <0,0001 |
| Control vs. LCP | **** | <0,0001 | GA vs. LCP | **** | <0,0001 |
| Control vs. AB | *** | 0,0003 | GA vs. AB | **** | <0,0001 |
| STP vs. GA | ns | 0,1580 | LCP vs. AB | **** | <0,0001 |

**Figure 2p.** Synergistic effects of different combinations of *G. acid, S. thermophilus*, and *L.casei*’s postbiotics on IL-33 expression in *H.pylori*-infected AGS cells.

| IL-33 | Summary | P value | IL-33 | Summary | P value |
| --- | --- | --- | --- | --- | --- |
| Control vs. AB | *** | 0,0009 | Control vs. GA+AB | ns | 0,9971 |
| Control vs. STP+GA | **** | <0,0001 | Control vs. STP+LCP+AB | * | 0,0311 |
| Control vs. STP+LCP+AB | **** | <0,0001 | Control vs. STP+GA+LCP+AB | **** | <0,0001 |
| Control vs. GA+LCP | **** | <0,0001 | Control vs. STP+GA+LCP+AB | ** | 0,0019 |

**Figure 2r.** Comparison of the effect of *G. acid*, *S. thermophilus*, and *L.casei*’s postbiotics on NF-kB expression level in the *H.pylori*-infected AGS cells with the antibiotic group.

| Nf-kB | Summary | Nf-kB | IL-33 | Summary | P value |
| --- | --- | --- | --- | --- | --- |
| Control vs. STP | **** | <0,0001 | STP vs. LCP | ns | >0,9999 |
| Control vs. GA | **** | <0,0001 | STP vs. AB | ns | >0,9999 |
| Control vs. LCP | **** | <0,0001 | GA vs. LCP | * | 0,0367 |
| Control vs. AB | **** | <0,0001 | GA vs. AB | * | 0,0480 |
| STP vs. GA | * | 0,0402 | LCP vs. AB | ns | 0,9998 |

**Figure 2s.** Comparison of the synergistic effect of different combinations of *G. acid*, *S. thermophilus*, and *L.casei*’s postbiotics with the antibiotic group on the level of Nf-kB expression in *H.pylori*-infected AGS cells.

| Nf-kB | Summary | P value | Nf-kB | Summary | P value |
| --- | --- | --- | --- | --- | --- |
| Control vs. AB | **** | <0,0001 | Control vs. GA+AB | **** | <0,0001 |
| Control vs. STP+GA | **** | <0,0001 | Control vs. STP+LCP+AB | **** | <0,0001 |
| Control vs. STP+LCP+AB | **** | <0,0001 | Control vs. STP+GA+LCP+AB | **** | <0,0001 |
| Control vs. GA+LCP | **** | <0,0001 | Control vs. STP+GA+LCP+AB | **** | <0,0001 |

**Figure 2t.** Comparison of gastric *H. pylori* bacterial load among treatment groups based on CFU counts (log₁₀ CFU/g): Demonstrating the impact of postbiotics, *Glycyrrhetinic acid*, and antibiotics.

| Groups | Summary | P value | Groups | Summary | P value |
| --- | --- | --- | --- | --- | --- |
| Negative control vs. Queternary | ns | >0,9999 | Queternary vs. Ternary | **** | <0,0001 |
| Negative control vs. Ternary | **** | <0,0001 | Queternary vs. Binary-1 | **** | <0,0001 |
| Negative control vs. Binary-1 | **** | <0,0001 | Queternary vs. Binary-2 | **** | <0,0001 |
| Negative control vs. Binary-2 | **** | <0,0001 | Queternary vs. Binary-3 | **** | <0,0001 |
| Negative control vs. Binary-3 | **** | <0,0001 | Ternary vs. Binary-1 | **** | <0,0001 |
| Infected control vs. Queternary | **** | <0,0001 | Ternary vs. Binary-2 | **** | <0,0001 |
| Infected control vs. Ternary | **** | <0,0001 | Ternary vs. Binary-3 | **** | <0,0001 |
| Infected control vs. Binary-1 | **** | <0,0001 | Binary-1 vs. Binary-2 | ns | 0,5009 |
| Infected control vs. Binary-2 | **** | <0,0001 | Binary-1 vs. Binary-3 | * | 0,0135 |
| Infected control vs. Binary-3 | **** | <0,0001 | Binary-2 vs. Binary-3 | ns | 0,3441 |

**
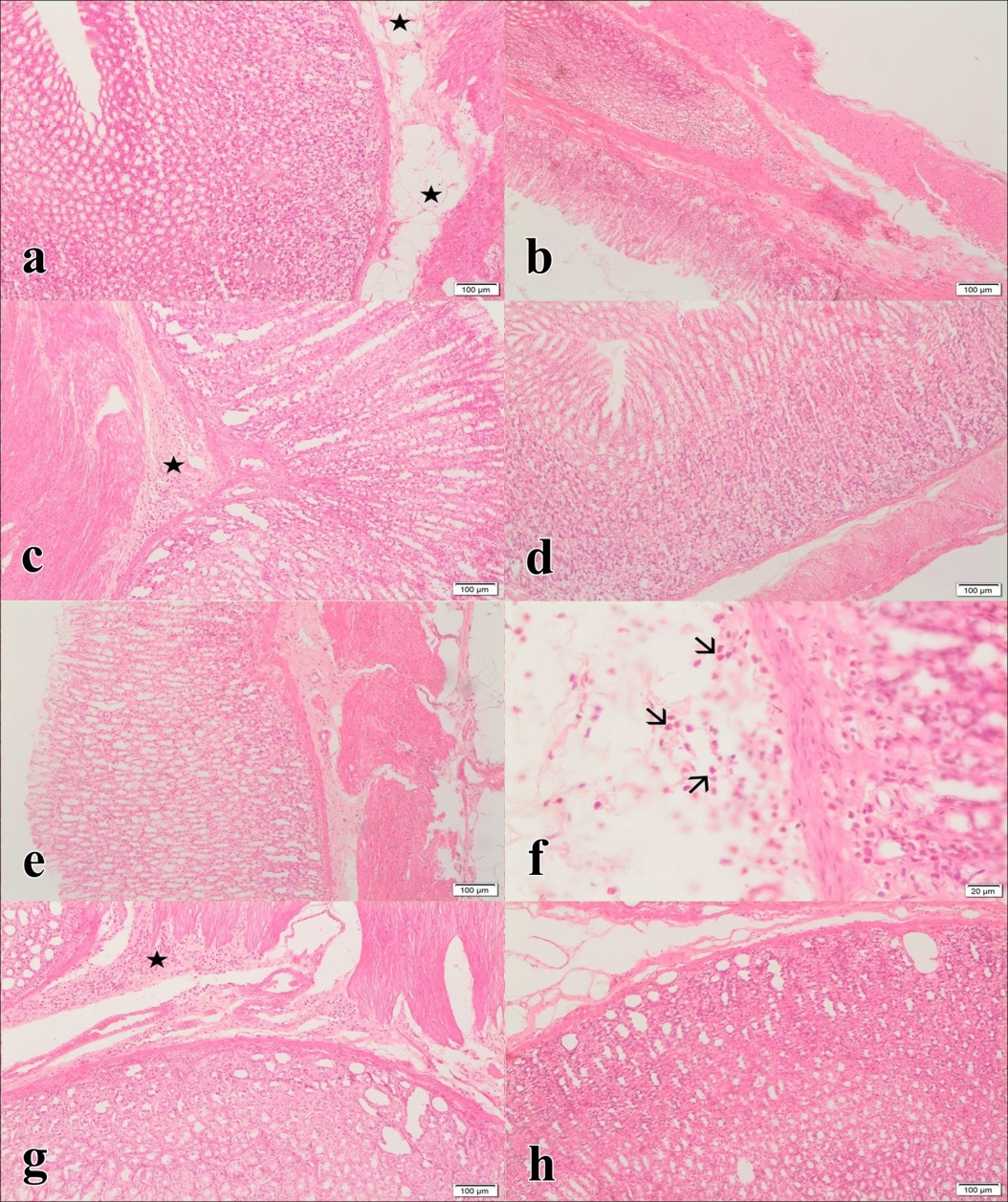
**

**Figure 20. a)** *S.thermophilus*’ postbiotics treatment group: Mild edema of the submucosa (stars), H×E. **b)** *G.acid* treatment group: Lymphoplasmocytic infiltration in the mucosa and submucosa, moderately severe infection, H×E. **c)** *L.casei*’s postbiotics treatment group: Mild lymphoplasmacytic infiltration in the submucosal region (star), H×E. **d)** Antibiotic treatment group, H×E. **e)** *S.thermophilus*’ postbiotics plus *G.acid* treatment group H×E. **f)** *S.thermophilus*’ postbiotics plus *Lactobacillus casei*’s postbiotics treatment group: Mild eosinophil leukocyte infiltration in the mucosal and submucosal region (arrows), H×E. **g)** *G. acid* plus *L.casei*’s postbiotics treatment group: Mild lymphoplasmacytic infiltration in the submucosa (star, star), H×E. **h)** *G. acid* plus antibiotic treatment group, H×E.


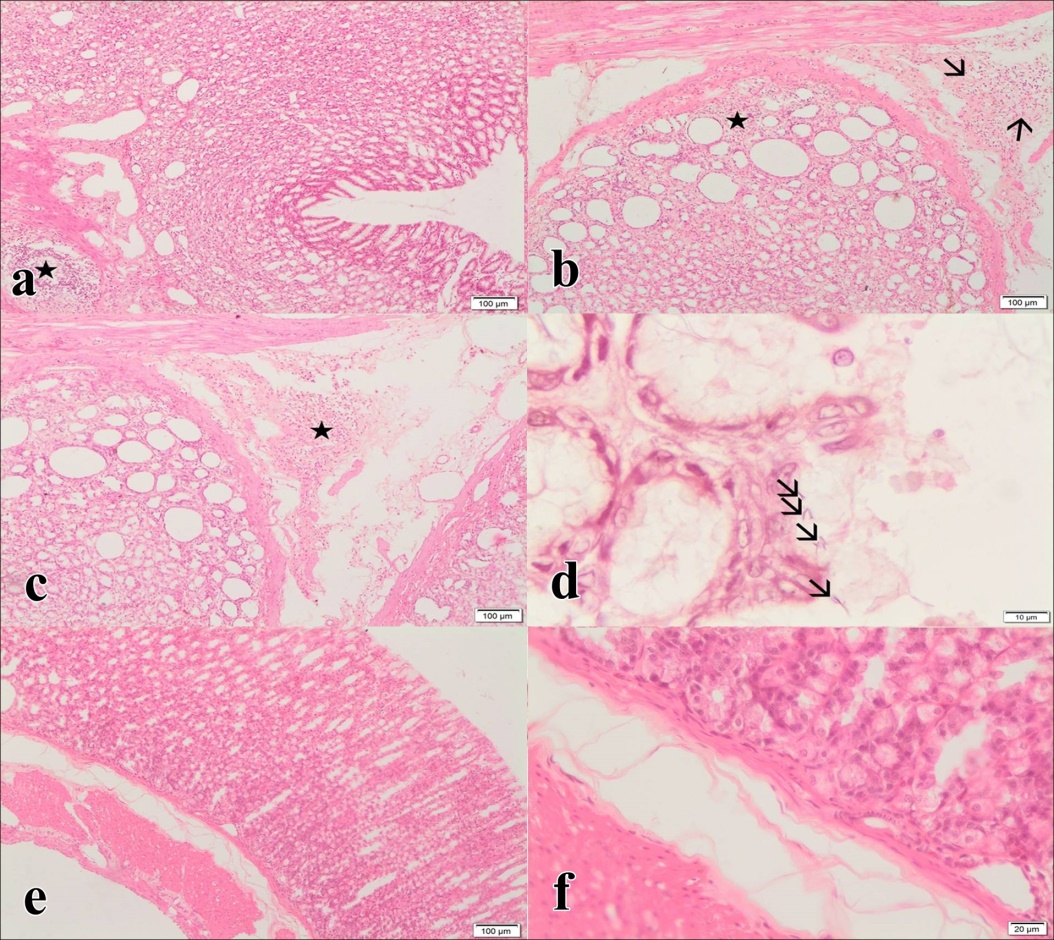


**Figure 21. a)** *S.thermophilus’* postbiotics plus *L.casei*’s postbiotics plus antibiotic treatment group: Moderate lymphoplasmacytic infiltration in the mucosal and submucosal region (star), H×E. **b)** *S.thermophilus*’ postbiotics plus *G. acid* plus *L.casei*’s postbiotics treatment group: Moderate lymphoplasmacytic infiltration in the mucosa (star) and submucosal region (arrows), H×E. **c)** *G. acid* plus *L.casei*’s postbiotics plus *S.thermophilus*’ postbiotics plus Antibiotic treatment group: Moderate lymphoplasmacytic infiltration in the mucosal and submucosal region (star), H×E. **d)** Infection control group. *H. pylori* colonization (arrows), 100×, H×E.**e)** Negative control group, H×E. **f)** Negative control group, H×E.
